# Disturbed sleep is associated with reduced verbal episodic memory and entorhinal cortex volume in younger middle-aged women with risk-reducing early ovarian removal

**DOI:** 10.3389/fendo.2023.1265470

**Published:** 2023-10-03

**Authors:** Nicole J. Gervais, Laura Gravelsins, Alana Brown, Rebekah Reuben, Mateja Perovic, Laurice Karkaby, Gina Nicoll, Kazakao Laird, Shreeyaa Ramana, Marcus Q. Bernardini, Michelle Jacobson, Lea Velsher, William Foulkes, M. Natasha Rajah, Rosanna K. Olsen, Cheryl Grady, Gillian Einstein

**Affiliations:** ^1^ Department of Psychology, University of Toronto, Toronto, ON, Canada; ^2^ Rotman Research Institute, Baycrest Health Sciences, Toronto, ON, Canada; ^3^ Groningen Institute for Evolutionary Life Sciences, University of Groningen, Groningen, Netherlands; ^4^ Cancer Clinical Research Unit, Princess Margaret Cancer Centre, University Health Network, Toronto, ON, Canada; ^5^ Genetics Program, North York General Hospital, Toronto, ON, Canada; ^6^ Department of Human Genetics, McGill University, Montreal, QC, Canada; ^7^ Lady Davis Institute, Segal Cancer Centre, Jewish General Hospital, Montreal, QC, Canada; ^8^ Departments of Psychiatry and Douglas Research Centre, McGill University, Montreal, QC, Canada; ^9^ Department of Psychology, Toronto Metropolitan University, Toronto, ON, Canada; ^10^ Tema Genus, Linköping University, Linköping, Sweden; ^11^ Women’s College Research Institute, Toronto, ON, Canada

**Keywords:** bilateral salpingo-oophorectomy, BRCA1/2, estradiol, sleep phenotype, verbal episodic memory, structural neuroimaging, entorhinal cortex

## Abstract

**Introduction:**

Women with early ovarian removal (<48 years) have an elevated risk for both late-life Alzheimer’s disease (AD) and insomnia, a modifiable risk factor. In early midlife, they also show reduced verbal episodic memory and hippocampal volume. Whether these reductions correlate with a sleep phenotype consistent with insomnia risk remains unexplored.

**Methods:**

We recruited thirty-one younger middleaged women with risk-reducing early bilateral salpingo-oophorectomy (BSO), fifteen of whom were taking estradiol-based hormone replacement therapy (BSO+ERT) and sixteen who were not (BSO). Fourteen age-matched premenopausal (AMC) and seventeen spontaneously peri-postmenopausal (SM) women who were ~10y older and not taking ERT were also enrolled. Overnight polysomnography recordings were collected at participants’ home across multiple nights (M=2.38 SEM=0.19), along with subjective sleep quality and hot flash ratings. In addition to group comparisons on sleep measures, associations with verbal episodic memory and medial temporal lobe volume were assessed.

**Results:**

Increased sleep latency and decreased sleep efficiency were observed on polysomnography recordings of those not taking ERT, consistent with insomnia symptoms. This phenotype was also observed in the older women in SM, implicating ovarian hormone loss. Further, sleep latency was associated with more forgetting on the paragraph recall task, previously shown to be altered in women with early BSO. Both increased sleep latency and reduced sleep efficiency were associated with smaller anterolateral entorhinal cortex volume.

**Discussion:**

Together, these findings confirm an association between ovarian hormone loss and insomnia symptoms, and importantly, identify an younger onset age in women with early ovarian removal, which may contribute to poorer cognitive and brain outcomes in these women.

## 1 Introduction

Sleep disturbance often precedes the diagnosis of Alzheimer’s disease (AD), is associated with cognitive impairments in patients with dementia (1) and importantly, is a modifiable risk factor (2). Reported in 25-66% of individuals with AD are sleep and circadian dysfunction, including sleep-wake cycle dysregulation (3, 4), insomnia (5, 6), and sleep apnea (7). This is particularly important for women, who experience more sleep disturbance and with greater adverse consequences than men (8). There are known sex differences in the rates of sleep disorders, with insomnia being more frequently diagnosed in women (9–13) and sleep apnea more common in men (14–16). Risk factors differ between women and men as well with chronic insufficient sleep increasing risk for hypertension in women, but not men, and habitually short and long sleep increasing risk for type 2 diabetes in women only (17). While fewer women have obstructive sleep apnea, higher risk for obstructive sleep apnea was associated with poorer episodic memory and executive function in women aged 45-59 years, whereas no such association was found in men of any age (18). Sleep disturbance among middle-aged women is common, with 40-60% in peri/early post-spontaneous menopause (SM) reporting sleep complaints (19). In a cohort of older women of mixed-menopause status, shorter sleep duration was associated with reduced verbal episodic memory (20). Our group has shown that women with early midlife bilateral salpingo-oophorectomy (BSO) have lower verbal episodic memory (21) and smaller hippocampal subfield volume (22). As such, sleep dysfunction is likely a critical issue for women’s cognitive health.

The prevalence of sleep disorders increases beginning at perimenopause and is associated with hormonal changes (7). Even among perimenopausal women without sleep disorders, hormone changes contribute to problems initiating and maintaining sleep. For example, increases in follicle stimulating hormone among women in perimenopause are associated with poorer sleep quality, more awakenings, and trouble sleeping (23–25), and on polysomnography recordings, are related to reduced sleep efficiency and increased waking after sleep onset (WASO) and sleep latency (26, 27).

While ovarian aging is the most common reason for entering menopause (spontaneous menopause, SM) and occurs at approximately 51 years, there are many menopauses (28). For example, when occurring prior to SM, bilateral oophorectomy results in the immediate loss of ovarian hormones, with long-term consequences that include more rapid cognitive decline (29) and increased risk for late-life dementia, including AD (30, 31). Women with early life ovarian removal have an even higher risk for both insomnia (23, 32) and sleep apnea (33) than women in SM.

Approximately 66% of all patients living with Alzheimer disease (AD) in the United States (34) are women, who also have greater overall lifetime risk (35), experience higher disease burden, including increased AD biomarkers (tau) in the medial temporal lobe (36, 37), and among those with another AD biomarker (beta amyloid), experience more rapid cognitive decline (38). These findings implicate reduced lifetime ovarian hormone exposure as a female-specific risk factor for AD, possibly by contributing to sleep dysfunction.

Reduced lifetime exposure to ovarian hormones, and particularly a younger age at menopause, is thought to contribute to the higher AD risk among women (39–43). Consistent with this, smaller volume in select subfields of the hippocampus, which is observed in the early stages of AD (44), is also associated with early ovarian removal (22). Sleep loss (45), including poorer sleep quality, lower efficiency, increased daytime sleepiness, higher arousal index, and longer duration of insomnia symptoms, are also associated with reduced hippocampal volume in mixed-sex cohorts (46, 47). Even one night of sleep deprivation increases beta amyloid accumulation in this region (48). Cortical structures within the medial temporal lobe are also affected by the early stages of AD (49), yet few have addressed the correspondence of anatomical changes to these regions with sleep disturbance. Since women with ovarian removal are more susceptible to insomnia (34), cognitive decline and AD later in life, we wondered whether insomnia risk is (1) detectable at early midlife and within a few years following BSO; (2) ameliorated by ERT; (3) more severe when compared to older women in SM, and (4) associated with structural changes to the medial temporal lobe and related functions. We addressed these questions in women who carry the breast cancer mutations, BRCA1 and 2, and who have had early midlife risk reducing BSO. Their sleep was measured using portable polysomnography to record brain activity during habitual sleep at their homes. Given that hormone therapy is associated with improved sleep in peri/post-SM women (50), and with larger hippocampal volume in women with early BSO (22), we stratified our cohort comparing groups based on whether they were currently taking estradiol-based hormone replacement therapy (ERT) or not (BSO). We also compared both BSO groups to age-matched premenopausal controls (AMC). We hypothesized that sleep disturbance consistent with insomnia risk (trouble initiating and maintaining sleep) would be detectable shortly after BSO. Since prior studies using polysomnography have already shown evidence for difficulty initiating and maintaining sleep in older women in peri/early post-SM, we wondered whether the sleep phenotypes of younger women with early BSO would resemble those of older SM women. Thus, an additional group comprising of women in peri/early post-SM were also included and their sleep compared to the BSO+ERT and AMC groups. None of these women in SM were taking hormone therapy and were therefore matched with the BSO group in the duration of hormone deprivation. We predicted that while both hormone deprived groups (BSO/SM) would show trouble initiating and maintaining sleep (indicated by a longer sleep latency and reduced sleep efficiency) relative to the BSO+ERT/AMCs groups, they would be similar to each other, indicating an earlier age of onset for sleep disturbance in women with early BSO. Finally, we explored the association between the sleep phenotype of these women and verbal episodic memory, which is sensitive to early ovarian removal (22), prodromal AD (51), and insomnia severity (52). We also correlated the sleep phenotypes with volumes of cortical medial temporal lobe structures and hippocampal subfields estimated using a manual segmentation approach on high-resolution T2-weighted MRI scans previously shown to be sensitive to prodromal AD (53) and early ovarian removal (22). Identifying such associations at midlife can enable a life-style intervention at the earliest point of change optimize treatment success in women at risk for late-life AD.

## 2 Methods

### 2.1 Participants

This cross-sectional analysis is part of a larger longitudinal study on the effects of early BSO on cognition and brain, which is described in detail elsewhere (21, 22). Briefly, thirty-one women with the *BRCA1/2* mutation (aged: 34-56) who had a prophylactic BSO at least 6 months before study onset and prior to the age of 49 years were recruited from local area hospitals. Fifteen were currently taking ERT (BSO+ERT), including six with concurrent micronized progesterone and four with Levonorgestrel. Sixteen women were not taking hormone therapy (BSO). The exclusion criteria included untreated health/psychiatric conditions, concussion with loss of consciousness, chemotherapy/radiation/adjuvant therapy for cancer within the past 6 months. Any MRI contraindication was an additional exclusion criterion for the MRI study portion. Additional exclusion criteria included hormonal contraceptive use or pregnancy/breastfeeding/fertility treatments within the past 6 months.

The AMC (*n*=14) and SM (*n*=17) groups were recruited from the community. Thirteen women in the SM group reported their last menstrual cycle to have been at least one year prior and so fit the *Stages of Reproductive Aging Workshop +10* criteria for SM (54). The remaining four women were in late perimenopause (final menstrual cycle >0.5 year<1 year).

Written informed consent was provided by all participants who received compensation for their time. All study procedures were approved by the Research Ethics Boards at the University of Toronto and McGill University.

### 2.2 Hormone assessment

Urine samples were provided either during the neuropsychological or scanning session and were processed for metabolites of estradiol (estrone glucuronide) and progesterone (pregnanediol-3 glucuronide) using enzyme-linked immunoassays described elsewhere (55). This permitted verification of the hormone status for the four groups.

### 2.3 Polysomnography recordings and sleep staging

Overnight polysomnography recordings were collected by the participants at their homes using a portable device (Vitaport-5/REMbo-234, *Temec Technologies*, The Netherlands). Although all participants were asked to record their sleep for three nights, scheduling challenges and occasional discomfort lead to some women having recorded their sleep for fewer nights; thus, the number of recordings per participant varied between one and three nights (M=2.38 SEM=0.19). The recordings included signals from two frontopolar electroencephalography electrodes (EEG; Fp1/Fp2), and two electrooculography electrodes (EOG; LOC/ROC), referenced to the left mastoid. A fifth signal, estimating facial electromyography from the other four signals, was also provided. These signals were digitized at 256 Hz, with a filter of 60 Hz.

All one hundred and eighty-five recordings were initially processed in 30-s epochs using *Z3score* (version 2.2.0, *Neurobit Technologies*) and automated sleep staging was acquired, which followed modified criteria from the American Academy of Sleep Medicine to account for two EEG electrodes (56, 57). Recordings were then visually inspected by trained experimenters (NJG, LG, AB, KL, GN) blind to group membership, who provided an overall quality rating (0-3) based on the availability and integrity of each EEG/EOG signal. Twenty-three recordings received the lowest rating and were subsequently excluded from further analysis. This resulted in fourteen participants having one recording removed and four with two recordings. The automated sleep staging for the remaining one hundred and sixty-two recordings was subsequently inspected and corrected by the experimenters. Since the automated sleep staging did not include cortical arousals, the experimenters manually scored them for each recording.

Sleep staging data were extracted from each recording using the *Wonambi* package for python (version 6.13, https://github.com/wonambi-python/wonambi). The following sleep parameters were then averaged across recordings for each participant: total sleep time, sleep efficiency (percentage of time in bed spent asleep), latency to the first sleep epoch (in min), percentage of total sleep time spent in each of the three stages of non-rapid eye movement sleep (N1-N3), and rapid eye movement sleep (REM), and wake after sleep onset (WASO, in min). The number of cortical arousals per hour of total sleep time (arousal index) was also averaged.

### 2.4 Subjective measures

A sleep diary was completed following each night of recorded sleep. Participants indicated their sleep and wake time, number of times they woke up at night, and how long on average it took them to fall back asleep. In addition, perceived sleep quality was rated on an 11-point scale (0 indicating the worst possible sleep quality, and 10 the best possible), Given that sleep disturbances can be related to nocturnal hot flashes (58), participants also indicated how many occurred on a given night and how bothersome they were using an 11-point scale (0 indicating not at all bothered and 10- extremely bothered). In addition, the Pittsburgh Sleep Quality Index (59) (PSQI), which assesses global sleep quality over the past month, was completed during an in-person session that included collection of medical history, demographic information, and neuropsychological testing. Mood measures were also completed during this session and include the Center for Epidemiological Studies - Depression Scale (60), which assesses depressive symptoms, and the Perceived Stress Scale (61), to assess stress at the time of testing. Higher scores on all these measures indicate poorer mood, stress, and sleep quality.

### 2.5 Structural image acquisition & segmentation of hippocampal subfields

High-resolution T2-weighted scans perpendicular to the long axis of the hippocampus (TR=3000ms, TE=66ms, 28 slices, voxel size= 0.4 x 0.4 x 3 mm, no skip, FOV=220 mm) were acquired using Siemens 3.0 T Prisma Scanner with a 32-channel head coil. These scans were viewed in native space using FSLView (v4.0.4) and manual segmentation of medial temporal lobe grey matter was performed separately for each hemisphere using to the Olsen-Amaral-Palombo protocol (62–65) by experimenters blind to group membership. The regions of interest (ROIs) were three hippocampal subfields (CA1, subiculum, and a region combining the dentate gyrus, CA2, and CA3; DGCA23), and four cortical structures (Perirhinal, Parahippocampal, anterolateral entorhinal cortex and posteromedial entorhinal cortices). Details related to image acquisition and volumetric analysis are described elsewhere (22).

### 2.6 Verbal episodic memory

Verbal episodic memory was assessed using Paragraph Recall [Logical Memory, Wechsler Memory Scale Form I (66)], which assesses short-term verbal memory (67). Participants were read a brief story before recalling details both immediately (immediate trial on Paragraph Recall, IMM) and approximately 30 minutes after presentation (delayed trial on Paragraph Recall, DEL). Correctly recalled verbatim details were scored (68), averaged and a percent retention score was calculated ((IMM-DEL)/IMM*100), with higher scores indicating poorer verbal episodic memory retention.

### 2.7 Statistical analysis

Statistical analyses were conducted using Statistical Package for Social Sciences v24 (IBM) and effect size estimates (η^2^ or Cohen’s d) were calculated for all parametric analyses. Statistical assumptions were tested, including normality and homogeneity of variance and when violated, outliers were corrected, or nonparametric tests were used. Follow-up comparisons for all analysis of variance or covariance (ANOVAs or ANCOVAs), and all correlations were corrected for false discovery rate (FDR).

We used ANOVAs to evaluate group differences on all cohort characteristics. This includes both hormone metabolites to confirm low ovarian hormone levels in both BSO and SM groups. Comparisons were also performed to ensure both BSO groups were similar in age to the AMC group, and that their age at menopause was significantly younger than those in the SM group. ANOVAs were also performed on the mood and subjective sleep measures, and on both the number and quality of polysomnography recordings to confirm the groups were well-matched.

ANCOVAs (or Kruskal-Wallis) with planned comparisons were performed on all sleep staging measures collected from polysomnography. These analyses controlled for quality ratings of the polysomnography recordings. To determine whether early BSO was associated with sleep disturbance, simple comparisons were made between the BSO and AMC groups. Comparisons were also performed between BSO+ERT and BSO to determine whether sleep disturbance is prevented by ERT. To confirm previous reports that women in SM experience sleep disturbance, we also compared the SM group to the AMC group. Finally, to explore whether sleep disturbance of women with early BSO is comparable to that observed in the older women in SM, we conducted comparisons between the BSO and SM groups. A separate set of analyses controlling for sleep diagnosis was also run to determine whether diagnosed sleep apnea had an impact on any observed group differences.

Since we have previously determined that medial temporal lobe volume and verbal episodic memory are reduced in women with early BSO (21, 22), our focus in the present study was on their association with sleep measures sensitive to early BSO. Sleep parameters showing significant group differences were selected before performing Pearson correlations (or Spearman correlations for measures that were not normally distributed) on the entire cohort as well as within each group. These were used to determine whether BSO-associated sleep disturbance may contribute to reduced brain health.

## 3 Results

### 3.1 Demographics

The cohort characteristics are shown in **Table 1**. As expected, groups differed significantly in age (*F* (3, 58) = 29.85, *p* = .000, η^2^ = .61), and age of menopause (*F* (2, 45) = 48.70, *p* = .000, η^2^ = .68). The SM group was significantly older than the other groups at both the time of testing (*p* = .000, *d* = 2.27-3.37) and the age of menopause (*p* = .000, *d* = 2.83-3.30). After winsorizing outliers detected in the hormone metabolite levels, the expected difference in the estradiol metabolite levels was observed (*F* (3, 55) = 6.75, *p* = .001, η^2^ = .27), with lower levels in the BSO and SM groups relative to AMC (BSO: *p* = .00 d =1.52; SM: *p* = .001 d =1.35) and BSO+ERT groups (BSO: *p* = .020 d =0.84; SM: *p* = .037 d = 0.72). Progesterone metabolite levels were not statistically different between groups. None of the other cohort characteristics differed significantly between groups, including mood or global sleep quality (**Table 1**).

**Table 1:** Participant characteristics by group (Mean ± SEM).

	AMC (*n* = 14)	BSO+ERT (*n* = 15)	BSO (*n* = 16)	SM (*n* = 17)
Urinary estrone-3-glucuronide (ng/ml)	36.93 ± 4.80	28.67 ± 4.76	16.83 ± 2.18^a,b^	18.22 ± 2.55^c,d^
Urinary pregnanediol glucuronide (mIU/ml)	2.45 ± 0.74	2.64 ± 0.92	0.90 ± 0.21	0.58 ± 0.09
Current Age (years)	43.64 ± 1.26	43.13 ± 1.09	46.00 ± 1.31	55.94 ± 0.81^c-e^
Education (years)	18.29 ± 0.65	18.20 ± 0.63	17.78 ± 0.61	16.29 ± 0.59
BMI (kg/m^2^)	23.56 ± 1.26	27.19 ± 1.18	25.21 ± 1.14	26.25 ± 1.10
Age at menopause (years)	--	39.87 ± 1.00	41.50 ± 0.97	51.35 ± 0.73^d,e^
Time since menopause (years)	--	3.59 ± 0.93	5.10 ± 0.93	4.55 ± 0.87
PSQI	4.05 ± 1.03	6.27 ± 0.84	6.64 ± 0.87	6.00 ± 0.94
CESD	10.79 ± 2.05	9.27 ± 1.98	8.81 ± 1.92	6.53 ± 1.86
PSS	15.64 ± 1.75	14.33 ± 1.69	13.25 ± 1.64	11.59 ± 1.59
Scanned participants	*n*=8	*n* =10	*n* =12	*n* =14
eTIV cm^3^	14865.71 ± 507.08	13574.66 ± 453.55	15194.79 ± 414.03	14346.18 ± 383.32
Scan image quality rating (0-3)	2.60 ± 0.18	2.60 ± 0.17	2.71 ± 0.15	2.32 ± 0.14

AMC, age-matched premenopausal controls; BMI, body mass index; BSO, bilateral salpingo-oophorectomy; CESD, Center for Epidemiological Studies Depression Scale; eTIV, estimated total intracranial volume; ERT, estradiol-based hormone replacement therapy; PSQI, Pittsburgh Sleep Quality Index; PSS, Perceived Stress Scale; SM, spontaneous menopause. ^a^BSO vs AMC, ^b^BSO vs BSO+ERT; ^c^SM vs AMC, ^d^SM vs BSO+ERT; ^e^SM vs BSO, p < .05. "-" denotes not applicable.

### 3.2 Sleep diary

No significant group differences were found for subjective measures of sleep or hot flashes during the polysomnography recordings (**Table 2**). Further, neither the number nor the quality of polysomnography recordings differed between groups (**Table 2**).

**Table 2:** Subjective sleep and hot flashes during polysomnography-recorded night (Mean ± SEM).

	AMC (*n* = 14)	BSO+ERT (*n* = 15)	BSO (*n* = 16)	SM (*n* = 17)
nighttime recordings (#)	2.07 ± 0.22	2.40 ± 0.21	2.50 ± 0.16	2.53 ± 0.15
Recording quality (1-3)	2.26 ± 0.15	2.02 ± 0.17	2.09 ± 0.16	1.84 ± 0.10
Perceived sleep quality/night (0-10)	5.14 ± 0.46	5.81 ± 0.45	6.02 ± 0.36	5.67 ± 0.49
Perceived total time in bed (hours)	5.47 ± 0.59	6.61 ± 0.50	6.24 ± 0.50	5.92 ± 0.46
Perceived awakenings (#)	2.30 ± 0.50	1.56 ± 0.28	2.40 ± 0.51	2.74 ± 0.46
Perceived average wake time between sleep bouts (min)	11.76 ± 3.56	18.92 ± 8.02	16.05 ± 2.99	9.84 ± 2.05
Nocturnal hot flashes reported (*n*)	0	2	10	6
Nocturnal hot flashes (#)/recording	--	1.34 ± 0.34	1.88 ± 0.72	2.06 ± 0.90
Severity of hot flashes (0-10)	--	2.67 ± 0.67	3.70 ± 0.52	4.25 ± 1.22
Sleep apnea (*n*)	0	3	0	1
Insomnia (*n*)	0	1	0	1

AMC, age-matched premenopausal controls; BSO, bilateral salpingo-oophorectomy; ERT, estradiol-based hormone replacement therapy; SM, spontaneous menopause. "-" denotes "not applicable", as in **Table 1**."#" denotes "number".

### 3.3 Polysomnography

As shown in **Table 3**, sleep latency was significantly different between groups (χ^2 =^ 13.91, p = .003), with the BSO and SM groups taking longer to fall asleep than the BSO+ET (BSO: Z = 2.57, p = .009; SM: Z = 2.83, p = .004) and AMC groups (BSO: Z = 2.25, p = .025; SM: Z = 2.62, p = .008). Groups also differed in sleep efficiency *F* (3, 62) = 4.48, *p* = .007, η^2^ = .19), which was lower for both the BSO and SM groups relative to BSO+ERT (BSO: *p* = .011, d = 0.95; SM: *p* =.002, d = 1.24), and for SM relative to AMC (*p* = 0.026, d =0.98). There were no other group differences on any of the other sleep measures (**Table 3**). Additional analyses controlling for sleep disorders resulted in the same pattern of results.

**Table 3:** Polysomnography sleep measures (Mean ± SEM), corrected for recording quality rating.

	AMC (*n* = 14)	BSO+ERT (*n* = 15)	BSO (*n* = 16)	SM (*n* = 17)
Total sleep time (hour)	6.36 ± 0.21	6.47 ± 0.20	6.56 ± 0.20	6.61 ± 0.20
Sleep efficiency (%)	90.01 ± 1.16	91.31 ± 1.10	87.29 ± 1.07^a^	86.37 ± 1.06^c,d^
Sleep latency (min)	12.56 ± 1.97	11.92 ± 2.62	27.35 ± 5.02^a,b^	33.78 ± 5.96^c,d^
%N1	7.92 ± 0.76	5.44 ± 0.72	6.53 ± 0.70	7.27 ± 0.69
%N2	64.17 ± 2.36	65.64 ± 1.30	66.27 ± 1.39	63.05 ± 0.99
%N3	5.03 ± 1.25	5.92 ± 1.19	5.75 ± 1.15	4.66 ±1.14
%REM	23.27 ± 1.30	22.95 ± 1.24	21.53 ± 1.20	24.66 ± 1.18
Arousal index (#arousals/hour)	4.61 ± 0.45	4.39 ± 0.42	4.93 ± 0.41	4.48 ± 0.40
WASO (min)	28.25 ± 4.06	25.60 ± 3.86	27.64 ± 3.74	29.54 ± 3.69

AMC, age-matched premenopausal controls; BSO, bilateral salpingo-oophorectomy; ET, estradiol-based hormone replacement therapy; SM =spontaneous menopause; WASO, wake after sleep onset. ^a^BSO vs BSO+ET, ^b^BSO vs AMC, ^c^SM vs AMC, ^d^SM vs BSO+ERT, SM vs BSO, BSO+ERT vs AMC; p < .05. "#" denotes "number" as in **Table 2**.

### 3.4 Correlations between polysomnography-derived sleep measures, medial temporal lobe volume, and verbal episodic memory

**Figure 1:**
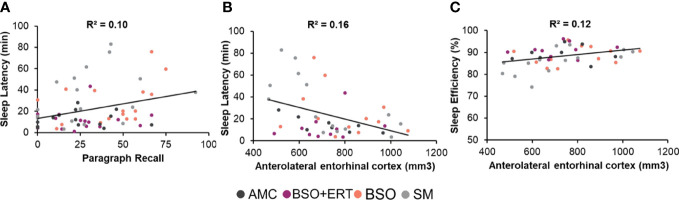
Scatterplots depicting significant associations within the entire cohort. Each point depicts a data point from a participant, with the colour corresponding to her group membership. **(A)** Positive association between the latency to enter the first sleep stage and the retention score for Paragraph Recall, with higher scores reflecting poorer verbal recall. **(B)** Negative correlation between the latency to enter the first sleep stage (Sleep latency in minutes) and the raw volume of the anterolateral entorhinal cortex (right hemisphere, mm^3^). **(C)** Positive association between the percent time in bed spent in a sleep stage (sleep efficiency) and the raw volume of the anterolateral entorhinal cortex (right hemisphere, mm^3^).

Across the entire cohort, longer sleep latency was associated with poorer retention on the Paragraph Recall (ρ = .28, *p* = .03; **Figure 1A**), and smaller volume of the right anterolateral entorhinal cortex (ρ = -.36, *p* = .015, **Figure 1B**). Sleep efficiency was positively associated with right anterolateral entorhinal cortex (ρ = .39, *p* = .008, **Figure 1C**). None of the other FDR-corrected correlations were significant. There were also significant within-group associations for AMC and SM groups; right anterolateral entorhinal cortical volume was negatively correlated with sleep latency (AMC: ρ = -.79, *p* = .02; SM: ρ = -.68, *p* = .007), and positively correlated with sleep efficiency (SM: ρ = .62, *p* = .019; all FDR-corrected).

## 4 Discussion

This is the first study to investigate whether objectively measured sleep disturbance contributes to reduced brain health in women on average five years from their early BSO. We used portable polysomnography across multiple nights and collected sleep metrics from each woman. While no subjective accounts of sleep disturbance (PSQI and sleep diaries) were found in women with early BSO, the polysomnography recordings revealed increased sleep latency and reduced sleep efficiency compared with age matched premenopausal women. ERT use among women with a BSO was associated with maintained sleep, as the sleep measures for those in the BSO+ERT group were comparable to the AMC group, and superior to the BSO group. Older women in SM also had increased sleep latency and reduced sleep efficiency relative to both AMC and BSO+ERT groups but were not significantly different than the BSO group. In other words, sleep in young middle-aged women resembled that of women in peri/early post-SM who were on average ten years older.

To identify consequences on cognitive and brain health, we then examined whether the two sleep measures sensitive to hormone deprivation were related to verbal episodic memory and medial temporal lobe volume. Poorer verbal episodic memory, which we previously found to be reduced in this cohort of women with BSO (21) was associated with taking longer to fall asleep (increased sleep latency), but was unrelated to sleep efficiency. Further, smaller right anterolateral entorhinal cortex volume was associated with both increased sleep latency and decreased sleep efficiency. Verbal memory impairments (69) and atrophy in the entorhinal cortex (70) are associated with the prodromal stage of AD. Thus, these findings suggest that within a few years following surgery and at early midlife, sleep disturbance experienced by women with BSO is comparable to that of older women in SM and may have important implications for cognitive and brain aging.

The superior sleep of women with a BSO taking ERT compared to those not taking ERT suggests that maintaining ovarian hormone levels may ameliorate early BSO-associated sleep disturbance. Better sleep has also been reported in peri/early post-SM women taking unopposed estradiol (71) or estrone (72), implicating estrogens in the beneficial effects on sleep. However, progestin and micronized progesterone therapy are also associated with improvements in sleep, including increased slow-wave (73, 74) and REM sleep (73) in SM women with sleep disturbances. Estradiol with and without progestin is also associated with improved polysomnography-measured sleep quality of women in SM with insomnia (75) and some studies report that micronized progesterone, alone, improves sleep quality (76). In the present study, estradiol was taken unopposed by most women (*n*=9) in the BSO+ERT group, but was combined with either a progestin, or micronized progesterone in the remaining six participants. Thus, it cannot be determined conclusively whether the beneficial effects of ERT are the result of estradiol alone or the combination with progestin/progesterone. It should be noted that not all studies report beneficial effects of hormone therapy, particularly when unspecified (77–81). When studies properly consider which types of hormone therapy are taken by their cohorts, the available data indeed suggests that sleep is improved among women in perimenopause or SM, and this is consistent with our findings in women with early BSO. However, greater clarity is needed on which hormones support good sleep and hence, women’s health. Future studies must not only adequately consider menopause type (SM or early BSO), but also adequately address different ERT regimens by directly compare those taking estradiol alone, progesterone or progestogens alone, to those taking a combined formulation.

In line with the beneficial effects of maintaining ovarian hormone levels via ERT on sleep in women with early BSO, studies in older women in peri/post SM show that changing hormone levels are correlated with poorer sleep. In one study, higher levels of FSH (which is inversely related to estradiol levels) were associated with reduced sleep efficiency and increased WASO in perimenopausal women without sleep complaints (26). A more recent study found that increases in sleep latency across a 10-year follow-up period were related to elevations in FSH, but not age (82). Changes in estradiol/FSH levels during perimenopause are associated with poorer sleep quality, more awakenings, and trouble sleeping (23–25). In a large cohort study of over 6000 women (Canadian Longitudinal Study on Aging), those in SM were more likely to require more than 30 minutes to fall asleep, and to meet criteria for sleep-onset insomnia disorder than women in pre/perimenopause, confirming that sleep disturbance persists into the postmenopausal period (83). The decreased sleep efficiency and increased sleep latency observed in the present study is also consistent with a sleep phenotype associated with insomnia (84), and has also been observed in patients with mild cognitive impairment (85). Thus, sleep disturbance associated with lower estradiol levels may manifest in difficulty initiating and maintaining sleep rather than in decreased total sleep time, or time in each sleep stage, and if persisting beyond early midlife, may become associated with cognitive decline.

While our findings are consistent with prior observations of SM-associated sleep disturbance, not all studies of middle-aged women in peri/post-SM or of mixed-menopause status report altered sleep phenotypes (78, 86). One possibility is that heterogeneity with respect to menopause may mask susceptibilities limited by type, or timing of menopause. This has been shown to lead to contrasting results in the cognitive literature (28). Another possibility is that the sleep phenotype that emerges soon after SM may be limited to insomnia, which is detected by some sleep measures, but not all. Consistent with this interpretation, one study showed comparable time spent in each sleep stage and in total sleep time of women in SM to those in pre/perimenopause, while cortical activity (i.e. higher beta power) was increased during NREM sleep (87).

Contrary to predictions, women with BSO who were not taking ERT did not demonstrate poorer objective sleep than those in SM. Instead, both groups, who were matched for duration of hormone loss showed similar patterns of sleep disturbance when compared to the AMC and the BSO+ERT groups. Together, these findings indicate that ovarian hormone loss at any age may contribute to sleep disturbance. Since the women with BSO are, on average, 10 years younger than those in SM, and age-related changes in sleep and circadian function are well-documented to begin at late midlife (88), one possibility is that early hormone loss via BSO shifts the decline in sleep typically observed around peri/early post-SM to an earlier age. Should sleep continue deteriorating in these women as they approach the age of SM, such disturbances will likely be worse than those in SM. In other words, the sleep of older women with early BSO may be more disturbed than age-matched women in SM. This is consistent with existing literature showing a higher risk for insomnia (23, 32) and sleep apnea (33) in older women who had their ovaries removed prior to menopause. The possibility for sustained or deteriorating sleep disturbance has important implications for dementia in women with early midlife BSO. Not only is sleep disturbance a risk factor for AD (2), women with early surgical menopause are already at higher risk for late-onset AD compared with women in SM (30, 31) and early life sleep disturbance may play a role in this increased risk. Future studies are needed to explore the trajectory of sleep decline in women who had their ovaries removed prior to SM and whether sleep disorders like insomnia elevate AD risk even further in these women. Such studies have important implications for early sleep interventions that may reduce AD risk.

It is possible that factors other than lower ovarian hormones may have contributed to poor sleep in the BSO and SM groups. Hot flashes are reported by 60-90% of women in perimenopause and in SM (89–91); they are more common in women in SM with insomnia (92) and correlate with sleep complaints, WASO, and reduced sleep efficiency (58, 93). This has led some to suggest that SM-associated sleep disturbance is a consequence of nighttime hot flashes (94), and the benefits of hormone therapy are often attributed to hot flash reduction (95). However, menopause-associated sleep disturbance also occurs independent of hot flashes (83, 96). In the present study, there were no differences in the frequency and severity of nocturnal hot flashes among groups who reported them (BSO, SM, BSO+ERT). Further, hot flash frequency and severity were not correlated with any sleep measure. Finally, while women with ovarian removal report more severe menopausal symptoms, including hot flashes (97), this was not the case in the present study. Therefore, it is unlikely that nocturnal hot flashes contributed meaningfully to the observed findings. Depressive symptoms have also been implicated in menopause-associated sleep disturbance (98, 99), yet in the present study, CES-D scores were similar across groups, and did not correlate with sleep measures. Taken together, the hormone deprivation-associated sleep disturbance in the present study was unlikely to be the result of increases in either nocturnal hot flashes or depressive symptoms.

We also found that within the entire cohort, longer sleep latency correlated with poorer verbal episodic memory, which we previously showed to be altered in women with early BSO irrespective of ERT (21). These findings are consistent with prior reports in mixed-sex studies, which show associations between verbal episodic memory and sleep latency (100), or deficits in episodic memory among patients with insomnia (101). It remains to be confirmed whether insomnia symptomatology contributes directly to memory reduction in young middle-aged women with early BSO. An alternative might be that insomnia symptomatology reduces verbal memory function in part, by altering hippocampal structure, which may subsequently lead to reduced verbal episodic memory (102). Insomnia severity is associated with smaller dentate gyrus and CA3 subfield volume of men (103), which we recently showed to be reduced in our cohort of women with early BSO^45^. Hippocampal subfields are highly sensitive to modulation by estrogens (104) and possess a high density of estrogen receptors. Thus, estradiol loss may act directly on hippocampal structure rather than indirectly via sleep disturbance to contribute to atrophy in this region. Consistent with this,. Consistent with this, we found no associations between sleep measures and hippocampal subfield volumes in the present study. However, reduced sleep efficiency and increased sleep latency were associated with smaller right anterolateral entorhinal cortical volume, a brain region with a much sparser density of estrogen receptors. Researchers have reported reduced cortical thickness in the parahippocampal-entorhinal of older women with early BSO, who were around 19 years post-surgery (105). Unfortunately, sleep was not assessed in that study and so it remains to be seen whether difficulty initiating sleep persists among older women with early BSO and if so, whether it contributes to atrophy in the cortical regions of the medial temporal lobe. Additionally, among individuals with beta-amyloid burden, greater tau deposition has been observed in the entorhinal cortex of older women than men (36). Sleep disturbances have also been shown to be related to entorhinal cortex volume in patients with Parkinson’s disease (106). Thus, should women with early BSO experience persistent sleep disturbance beyond the first few years after surgery, one implication might be increased tau burden and atrophy in the entorhinal cortex later in life, consistent with their elevated AD risk. Importantly the anterolateral entorhinal cortex is one of the earliest cortical regions affected by AD pathology (49). Thus, future research should determine whether this early midlife association between sleep disturbance, verbal episodic memory, and anterolateral entorhinal cortex volume leads to greater tau deposition and AD in later life.

### 4.1 Strengths and limitations

There are a number of important limitations to the present study, including the cross-sectional design, small cohort size, and heterogeneous ERT regimens, all of which limit firm conclusions about whether early midlife hormone deprivation is directly responsible for the observed sleep disturbances. Additionally, our cohort did not include women in SM who were taking ERT, preventing us from determining whether ERT benefits sleep in these women. Further, our hormonal assessments excluded FSH, which others have shown to be associated with sleep disturbance. As well, volumetric estimates did not include brain regions involved in regulating the sleep-wake cycle, such as the suprachiasmatic nucleus, limiting our understanding of how ovarian removal contributes to sleep disturbance in these women. Despite these limitations, our study includes some notable strengths. We included a homogeneous group of women with BRCA1/2 who experienced early and abrupt ovarian hormone loss. The majority of studies examining menopause-associated sleep disturbance fail to consider menopause type despite differences in sleep disturbance risk. While our study did not detect differences between the two menopause types, the age difference between the two groups indicates that sleep is disturbed in women with BSO at an earlier age. Since they will live for longer with persistently low ovarian hormones than women in SM, sleep disturbance may be extended, potentially worsening memory and brain changes. An additional benefit of the present study was the assessment of sleep physiology using a portable polysomnography device. Not only is this the first study of women with early BSO to assess sleep using objective methods this approach permitted collection of sleep recordings across multiple nights at the participants’ homes, providing a closer approximation of their habitual sleep patterns than would be observed from a laboratory setting (107). Finally, by matching the BSO and SM groups on duration of ovarian hormone loss, we were able to determine that the earliest sleep disturbances in younger women with BSO, are comparable to those of women approximately 10 years older.

### 4.2 Conclusion

This is the first study to demonstrate early midlife sleep disturbance in women with early ovarian removal, who are at risk for late-life AD. The disturbances were restricted to sleep latency and sleep efficiency, were not observed in those who were taking ERT, and were comparable to those of older women in SM who were matched in terms of duration of hormone deprivation. The sleep phenotype of the two hormone deprived groups is consistent with insomnia symptomatology and implicates ovarian hormone deprivation, not age, at early midlife. Further, in all groups, these symptoms were related to poorer verbal episodic memory and smaller right anterolateral entorhinal cortical volume, both of which are affected in the prodromal stages of AD. Future prospective studies are needed to determine whether early midlife sleep disturbance persists beyond the first few years following BSO and contributes to dementia risk later in life. Should this be the case, interventions targeting early midlife sleep disturbance may prove to benefit both sleep health and brain aging.

## Data availability statement

The raw data supporting the conclusions of this article will be made available by the authors, without undue reservation.

## Ethics statement

The studies involving humans were approved by Research Ethics Boards at the University of Toronto and McGill University. The studies were conducted in accordance with the local legislation and institutional requirements. The participants provided their written informed consent to participate in this study.

## Author contributions

NG: conceptualization, data curation, formal analysis, funding acquisition, investigation, project administration, resources, visualization, and writing – original draft. LG: data curation, investigation, methodology, and writing – review & editing. AB: data curation, investigation, methodology, and writing – review & editing. RR: writing – review & editing and investigation. MP: investigation and writing – review & editing. LK: writing – review & editing, investigation and project administration. GN: investigation and writing – review & editing. KL: writing – review & editing and investigation. SR: investigation and writing – review & editing. MB: writing – review & editing and resouces. MJ: writing – review & editing and resouces. LV: writing – review & editing and resouces. WF: writing – review & editing and resouces. MR: writing – review & editing. RO: writing – review & editing, methodology and supervision. CG: supervision and writing – review & editing. GE: conceptualization, methodology, supervision, writing – review & editing.
